# SNPs Altering Ammonium Transport Activity of Human Rhesus Factors Characterized by a Yeast-Based Functional Assay

**DOI:** 10.1371/journal.pone.0071092

**Published:** 2013-08-13

**Authors:** Aude Deschuyteneer, Mélanie Boeckstaens, Christelle De Mees, Pascale Van Vooren, René Wintjens, Anna Maria Marini

**Affiliations:** 1 Biologie du Transport Membranaire, Université Libre de Bruxelles, Gosselies, Belgium; 2 Laboratoire des Biopolymères et des nanomatériaux supramoléculaires, Université Libre de Bruxelles, Brussels, Belgium; Newcastle University, United Kingdom

## Abstract

Proteins of the conserved Mep-Amt-Rh family, including mammalian Rhesus factors, mediate transmembrane ammonium transport. Ammonium is an important nitrogen source for the biosynthesis of amino acids but is also a metabolic waste product. Its disposal in urine plays a critical role in the regulation of the acid/base homeostasis, especially with an acid diet, a trait of Western countries. Ammonium accumulation above a certain concentration is however pathologic, the cytotoxicity causing fatal cerebral paralysis in acute cases. Alteration in ammonium transport via human Rh proteins could have clinical outcomes. We used a yeast-based expression assay to characterize human Rh variants resulting from non synonymous single nucleotide polymorphisms (nsSNPs) with known or unknown clinical phenotypes and assessed their ammonium transport efficiency, protein level, localization and potential trans-dominant impact. The HsRhAG variants (I61R, F65S) associated to overhydrated hereditary stomatocytosis (OHSt), a disease affecting erythrocytes, proved affected in intrinsic bidirectional ammonium transport. Moreover, this study reveals that the R202C variant of HsRhCG, the orthologue of mouse MmRhcg required for optimal urinary ammonium excretion and blood pH control, shows an impaired inherent ammonium transport activity. Urinary ammonium excretion was *RHcg* gene-dose dependent in mouse, highlighting MmRhcg as a limiting factor. HsRhCG^R202C^ may confer susceptibility to disorders leading to metabolic acidosis for instance. Finally, the analogous R211C mutation in the yeast ScMep2 homologue also impaired intrinsic activity consistent with a conserved functional role of the preserved arginine residue. The yeast expression assay used here constitutes an inexpensive, fast and easy tool to screen nsSNPs reported by high throughput sequencing or individual cases for functional alterations in Rh factors revealing potential causal variants.

## Introduction

The ammonia molecule (NH_3_) is in constant equilibrium with the charged form ammonium (NH_4_^+^), the latter representing the most abundant part at classical physiological pH values. Hereafter, we will mainly use the term ‘ammonium’ to refer to the sum of these two molecules, unless a distinction is necessary. Ammonium is ubiquitous on earth and constitutes an important nitrogen source for microorganisms and plants, while it is primarily documented for the cytotoxic consequences of its accumulation in animals [1]. Ammonium is mainly produced during protein catabolism and by the activity of the intestinal flora. The liver plays a major role in detoxifying the molecule into urea and glutamine, preventing its accumulation in the organism. In parallel, renal ammonium production and subsequent urinary elimination constitute an important process to maintain acid/base homeostasis [2]. The process is even more capital upon acid challenge, as generally happens with a classical Western acid diet [3], [4]. For decades the transmembrane transport of ammonium was recognized as mainly relying on passive diffusion of the neutral form NH_3_ [5]. Whether specific ammonium transport systems exist and whether these could play important patho-physiological functions remained open questions since the early nineties.

Using functional complementation in *Saccharomyces cerevisiae*, the first genes encoding specific ammonium transport systems from yeast (ScMep1) and plant *Arabidopsis thaliana* (AtAmt1;1) were simultaneously cloned, leading to the identification of a novel protein family named Mep-Amt [6], [7]. We next revealed that the mammalian Rhesus factors, responsible for the Rh blood group immunogenic reactions but still functional orphans, also belonged to this new, enlarged, Mep-Amt-Rh superfamily [8]. The human erythroid HsRhAG factor and a kidney homolog, HsRhCG, proved functional upon expression in yeast, both conferring bidirectional ammonium transport to a strain defective in endogenous ammonium transport systems [9], [10]. The crystal structures of a few members of this family, including human HsRhCG protein, were in the meantime solved, revealing a trimeric association, each monomer forming a putative conducting pore in its center [11]–[16]. The mechanism of gating control of Rh proteins remains unknown. A particular mutation in the C-terminal cytosolic extension of yeast Mep proteins was shown to lead to inactive forms able to poison the activity of coproduced native monomers in the multimeric complex via trans-inhibitory effects [17]. Similar allosteric interactions were observed in plant and other fungal Mep-Amt complexes leading to the proposal of a functional role of a conserved portion of the Mep-Amt C-terminal extension in the control of the pore gating [18]–[21]. As the C-terminal extension is not conserved when comparing Mep-Amt to Rh proteins, a similar gating role of the C-terminus of Rh proteins remains to be evaluated.

The RhCG protein is present at the surface of epithelial cells, including the renal collecting duct cells involved in urinary ammonium excretion [22]–[24]. Phenotypic analysis of mice lacking MmRhcg shows that this protein is necessary to maximal urinary ammonium excretion by altering the transepithelial ammonia permeability of collecting ducts. MmRhcg is required to maximal urinary acidification, its absence causing metabolic acidosis and leading to lethality according to the intensity of the applied acid stress [25]. For instance, mice lacking MmRhcg have features of incomplete “distal renal tubular acidosis” (dRTA), a human pathology comprising hereditary forms whose determinants are not completely known [26]. MmRhcg also localizes to the apical membrane of epididymal cells, its absence being accompanied by a decrease in the epididymal fluid pH. MmRhcg likely plays a role in the maintenance of the homeostatic conditions not only in the kidney but also in the male reproductive tract [25]. Hence, particular polymorphisms in human *RHCG* could be associated to pathological outcomes.

Of note, overhydrated hereditary stomatocytosis (OHSt), a rare dominantly-inherited haemolytic anemia, characterized by leakage of important monovalent cations (K^+^, Na^+^) at the red cells membrane, was recently associated to two mutations in the human *RHAG* gene leading to the I61R and F65S substitutions [27]. These mutations were proposed to confer monovalent cation conductance to the HsRhAG protein. Subsequent studies revealed that while HsRhAG^F65S^ showed an impaired efficiency in accumulating methylamine, both native and HsRhAG^F65S^ human proteins had an influence on the activation of endogenous cation transport pathways in a heterologous xenopus model [28]. A reduced ammonia-induced alkalinisation was observed in erythrocytes expressing HsRhAG^F65S^ [29]. Altogether, these studies indicate that HsRhAG^F65S^ has an impaired amine transport activity, but these also reveal the difficulty to distinguish direct effects from secondary consequences of the OHSt mutations.

Genome-wide association studies highlight DNA variants which could directly or indirectly contribute to clinical phenotypes. In addition, direct sequencing of the human genome via high throughput technologies is also affording the potential to uncover causal variants, the challenge being to develop fast and easy tools to screen these single nucleotide polymorphisms (SNPs) for potential functional alterations. The dbSNP NCBI database of SNPs (http://www.ncbi.nlm.nih.gov/SNP/) reports a number of non synonymous SNPs (nsSNPs) in human *RH* genes, including *RHCG*, with unknown functional or clinical impacts.

Here, we used the yeast *Saccharomyces cerevisiae* as an expression tool to characterize human Rh variants resulting from nsSNPs with known or unknown clinical outcome and assess the consequences of these mutations on the ammonium transport function. We show that the HsRhAG^I61R^ and HsRhAG^F65S^ variants associated to OHSt are altered in bidirectional ammonium transport. From the characterization of three HsRhCG variants, we identify HsRhCG^R202C^ as inherently impaired in bidirectional ammonium transport. We show that MmRhcg is a limiting factor for urinary ammonium excretion in mice. Structure-function analyses highlight Arg202 of HsRhCG and its Arg211 counterpart in yeast ScMep2 as playing an important and likely conserved role in the function of Mep-Amt-Rh proteins. HsRhCG^R202C^ may confer susceptibility to disorders linked to acid/base homeostasis or ammonium detoxification.

## Results

### Characterization of nsSNPs altering inherent bidirectional ammonium transport via HsRhAG and HsRhCG

**Figure 1:**
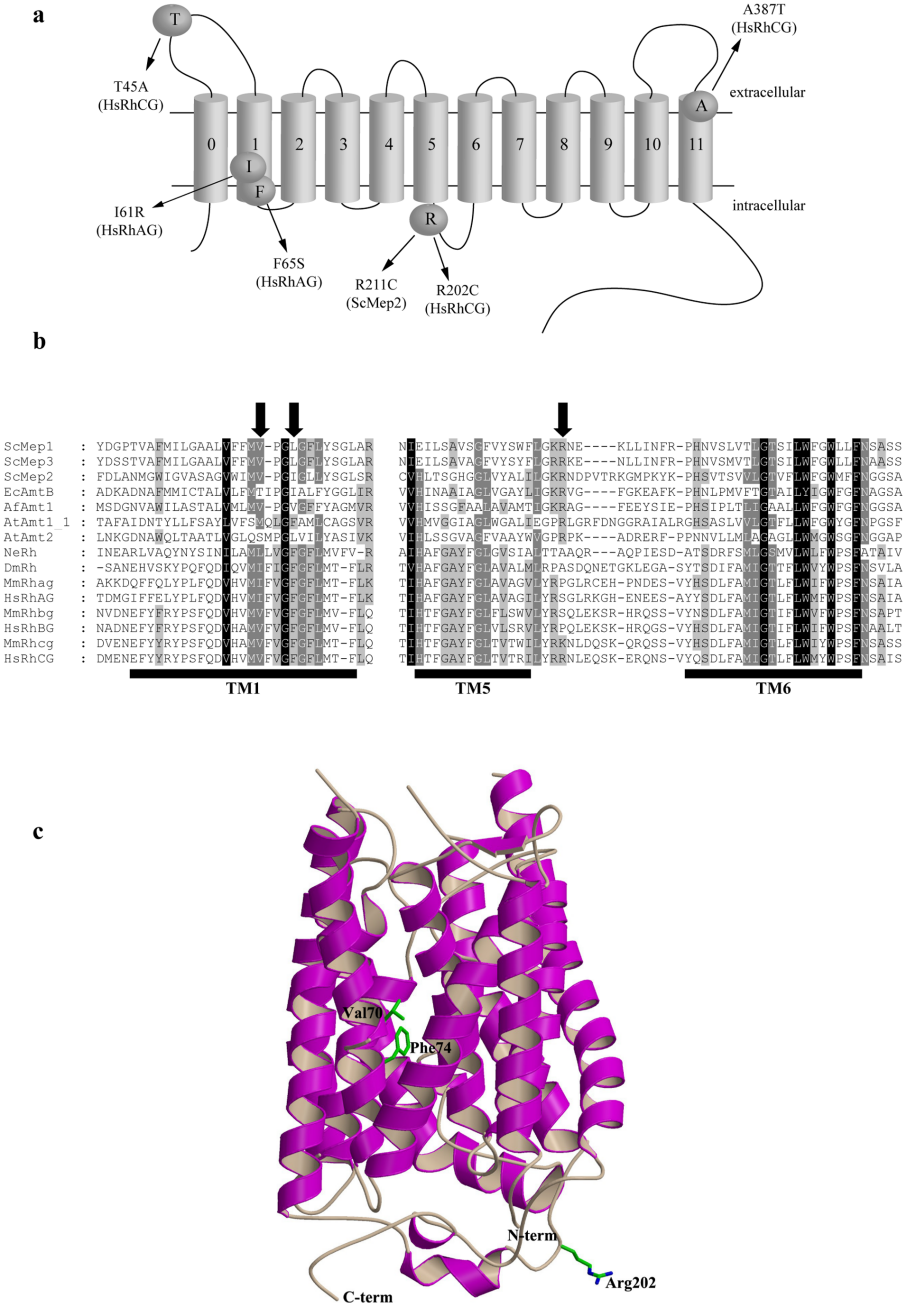
Location of the considered amino acid substitutions in HsRhAG, HsRhCG, and ScMep2. **a** Schematic representation of the predicted topology of Mep-Amt-Rh proteins. The first transmembrane segment (0) is absent in ScMep2 protein. **b** Partial primary sequence alignment of Mep-Amt-Rh proteins from different organisms. SWISS PROT or GeneBank accession numbers are referred hereafter. ScMep1, 2, 3: *S. cerevisiae* Mep1 (P40260), Mep2 (P41948), and Mep3 (P53390); EcAmtB: *E. coli* AmtB (P69681); AfAmt1: *A. fulgidus* Amt1 (O29285); AtAmt1_1, 2: *A. thaliana* Amt1;1 (P54144) and Amt2 (Q9M6N7); NeRh: *N. europaea* Rh50 (Q82X47); DmRh: *D. melanogaster* Rh (Q9V3T3); MmRhag, bg, cg: *M. musculus* Rhag (Q9QUT0), Rhbg (Q8BUX5) and Rhcg (Q9QXP0); HsRhAG, BG, CG: *H. sapiens* RhAG (Q02094), RhBG (AAL05978) and RhCG (Q9UBD6). The limits of transmembrane helices (TM) 1, 5 and 6 of human HsRhCG are deduced from the crystal structure of this protein (pdb code 3HD6) and are underlined. The location of Ile61 and Phe65 of HsRhAG and Arg202 of HsRhCG is indicated by an arrow. **c** Ribbon representation of the HsRhCG X-ray structure with labelled and depicted Val70, Phe74 and Arg202 residues.

Monomers of Mep-Amt-Rh proteins are made of 11 transmembrane helices (TM1 to TM11), like in Amt and yeast Mep proteins, or 12 transmembrane helices, with an additional helix at the N-terminus (termed TM0), like in mammalian Rh proteins (Fig. 1a). The I61R and F65S mutations in HsRhAG are the consequence of two polymorphisms associated with overhydrated hereditary stomatocytosis [27]. By sequence and structural similarities with HsRhCG [16], both HsRhAG residues are predicted to lie in the transmembrane helix TM1 (Fig. 1b). I61 and F65 in HsRhAG correspond to V70 and F74 in HsRhCG, respectively. In the HsRhCG structure (pdb code 3HD6), both residues overlap in their positioning and are oriented towards the centre of the putative conducting pore (Fig. 1c). At the analogous position of F65 in HsRhAG, a Phe is also found in other Rh proteins, including in the bacterial NeRh protein, while Ile and Val, thus smaller residues, are more usually found in Mep-Amt proteins like in EcAmtB and AfAmt1, respectively (Fig. 1b and data not shown). The NCBI database reports about 42 missense mutations in exons of human *RHCG*. We tested the functional impact of 3 nsSNPs originally available for *RHCG* (Table 1). One of these *RHCG* variants (rs117284582) leads to the T45A substitution in HsRhCG in the first extracellular loop (Fig. 1a), just before the likely site of N-glycosylation ^48^NLS^50^ [30]. The rs17807723 variant leads to the R202C substitution in the HsRhCG protein and is the nsSNP with the highest Grantham value in this study (Table 1). In the crystal structure of HsRhCG, Arg202 is located in the cytosolic 15-residue long loop linking the transmembrane helices TM5 and TM6 (Fig. 1a, 1b and 1c). The rs112948665 variant leads to the A387T substitution in the beginning of the last TM helix of HsRhCG (Fig. 1a).

**Table 1:** List of non synonymous single nucleotide polymorphisms in *RHAG* and *RHCG* genes analyzed in this study.

Gene	dbSNP rs# cluster id	Amino acid position	Amino acid change	Grantham value (D)	Minor allele frequence (MAF)	Clinical significance	Reference
*RHAG*	/	61	Ile → Arg	98	NA	Yes	[27]
*RHAG*	/	65	Phe → Ser	155	NA	Yes	[27]
*RHCG*	rs117284582	45	Thr → Ala	58	0.022	Unknown	/
*RHCG*	rs17807723	202	Arg → Cys	180	0.090	Unknown	/
*RHCG*	rs112948665	387	Ala → Thr	58	NA	Unknown	/

NA  =  not available; MAF Source  = 1000 Genome.

**Figure 2:**
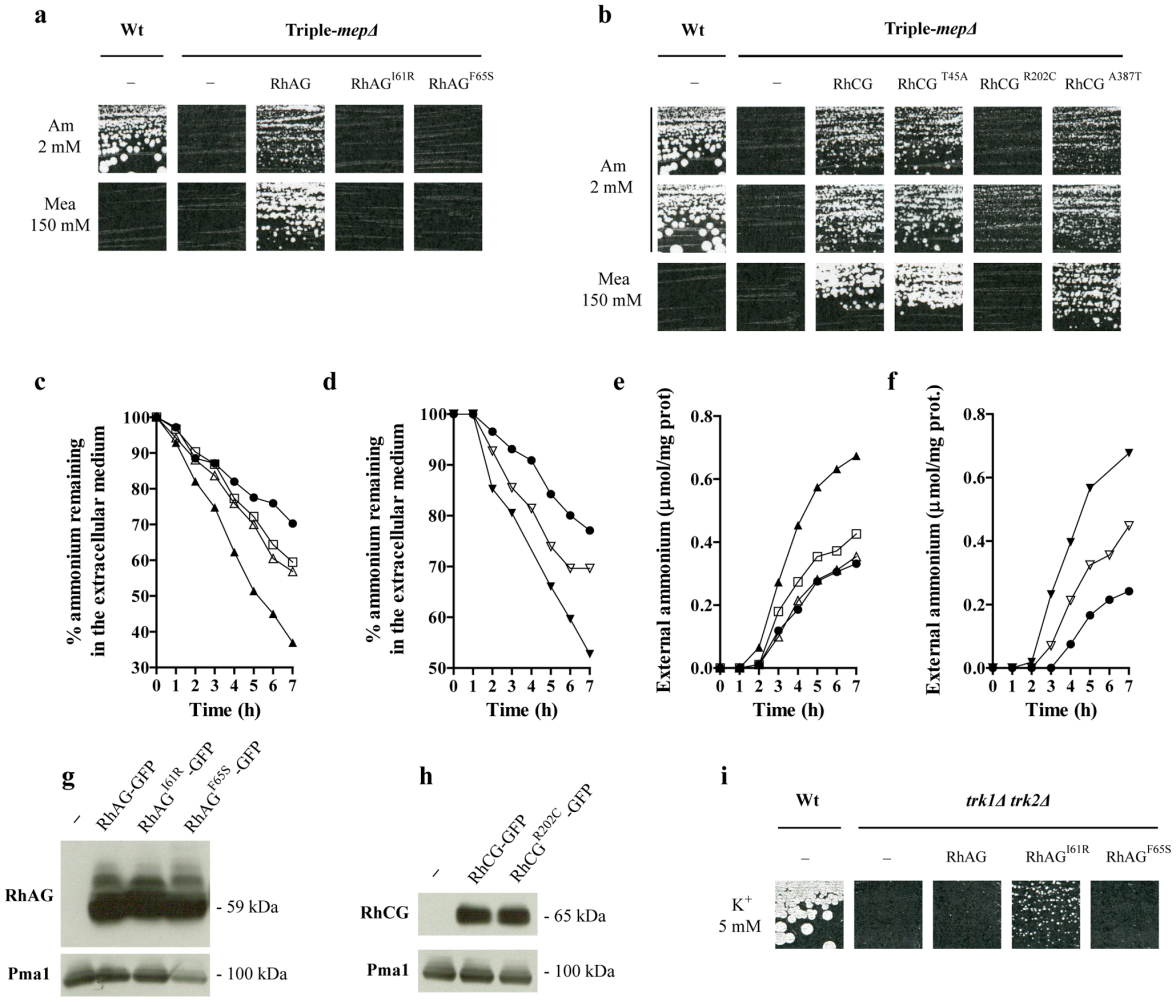
OHSt-related HsRhAG variants and HsRhCG^R202C^ are altered in inherent bidirectional ammonium transport. **a–b** Growth tests on solid minimal medium containing as the sole nitrogen source 2 mM ammonium (Am 2 mM), or proline supplemented with a toxic concentration of methylammonium (Mea 150 mM). Wild-type cells (23344c) were transformed with the empty p426 vector (−), and triple-*mepΔ* cells (31019b) were transformed with the empty p426 vector (−), or with a multi-copy plasmid bearing the native (HsRhAG, HsRhCG) or the mutated *HsRHAG* and *HsRHCG* genes (HsRhAG^I61R^, HsRhAG^F65S^, HsRhCG^T45A^, HsRhCG^R202C^, HsRhCG^A387T^). Cells were incubated for 7 days (a), and for 7 days (Mea 150 mM, Am 2 mM upper line) or 14 days (Am 2 mM bottom line) (b), at 29°C. **c-d** Ammonium removal assays. Triple-*mepΔ* cells (31019b) were transformed with p426 (*•*), p426-HsRhAG (*▴*), p426-HsRhAG^I61R^ (*Δ*), p426-HsRhAG^F65S^ (*□*), p426-HsRhCG (▾) or p426-HsRhCG^R202C^ (∇). **E–f** Ammonium excretion assays. Same cells used in (c) and (d). **g–h** Immunodetection of the HsRhAG-GFP and HsRhCG-GFP variants expressed in yeast. Triple-*mepΔ* cells (31019b) were transformed with the empty p426 vector (−), or with a multi-copy plasmid bearing GFP-tagged native (HsRhAG-GFP, HsRhCG-GFP) or mutated *HsRHAG* and *HsRHCG* genes (HsRhAG^I61R^-GFP, HsRhAG^F65S^-GFP, HsRhCG^R202C^-GFP). Membrane-enriched cell extracts were prepared from cells grown on glutamate minimal medium, separated by SDS-PAGE and immunoblotted with anti-GFP antibodies. ScPma1 was immunodetected as a loading control. **i** Growth tests of yeast strains on solid YNB medium with glutamine as the sole nitrogen source and supplemented with 5 mM KCl (K^+^ 5mM). Wild-type (S288c) and *trk1Δ trk2Δ* cells (CY162) were transformed as in (a) and incubated for 5 days at 29°C.

We used yeast as an expression tool to test the ammonium transport function of the reported HsRhAG polymorphisms and to eventually uncover human polymorphisms leading to HsRhCG proteins with altered function. The amino acid substitutions were introduced in Rh proteins using site-directed mutagenesis on yeast-based multi-copy vectors allowing the expression of native or GFP-tagged versions of the human Rh factors [9], [10]. Yeast cells deprived of their three endogenous ammonium transport systems, ScMep1, ScMep2 and ScMep3 (hereby termed ‘triple-*mepΔ*’ cells) are unable to grow when low ammonium concentrations are provided as sole nitrogen source [31]. Unlike native HsRhAG, expression of HsRhAG^I61R^ and HsRhAG^F65S^ did not allow growth of triple-*mepΔ* cells on a medium containing 2 mM ammonium indicating that the OHSt-related variants are altered in ammonium transport (Fig. 2a). While HsRhCG^T45A^ and HsRhCG^A347T^ behaved as native HsRhCG, expression of the HsRhCG^R202C^ variant did not allow growth of triple-*mepΔ* cells in presence of 2 mM ammonium indicating that this protein has altered ammonium transport efficiency (Fig. 2b). After prolonged incubation of the cells, growth of the triple-*mepΔ* strain expressing HsRhCG^R202C^ was detectable, suggesting that the latter variant has conserved some residual activity (Fig. 2b).

Consistent with altered ammonium transport, ammonium removal assays in liquid medium showed that triple-*mepΔ* cells expressing neosynthesized HsRhAG^I61R^, HsRhAG^F65S^ or HsRhCG^R202C^ are impaired in their ammonium removal efficiency (Fig. 2c and 2d). These data indicate that HsRhAG^I61R^, HsRhAG^F65S^ and HsRhCG^R202C^ proteins have a reduced ammonium transport function.

We previously showed that Rh factors are able to mediate ammonium transport in a bidirectional way [9]. Their expression enhanced the ammonium excretion rate of triple-*mepΔ* cells growing on a medium supplied with arginine, a nitrogen source whose catabolism leads to intracellular ammonium production [9]. In contrast to native HsRhAG or HsRhCG, expression of neosynthesized HsRhAG^I61R^, HsRhAG^F65S^ or HsRhCG^R202C^ allowed a reduced efficiency of ammonium excretion by triple-*mepΔ* cells, indicating that the mutated proteins are altered in their ability to export ammonium (Fig. 2e and 2f).

The HsRhAG and HsRhCG proteins were shown to confer to triple-*mepΔ* cells a resistance to high concentrations of methylammonium, a toxic analogue of ammonium, a phenotype possibly reflecting an increased efficiency in methylammonium excretion [9]. In contrast to native HsRhAG and HsRhCG, the HsRhAG^I61R^, HsRhAG^F65S^ and HsRhCG^R202C^ variants were unable to confer resistance to methylammonium to triple-*mepΔ* cells (Fig. 2a and 2b).

Western blot analysis with the GFP-tagged versions of the HsRhAG and HsRhCG variants were performed to test whether the alteration of the ammonium transport function could be due to defective protein levels. Growth tests showed that the GFP-tag did not perturb the phenotype conferred by the HsRhAG and HsRhCG variants (data not shown). Immunodetection of HsRhAG^I61R^-GFP, HsRhAG^F65S^-GFP and HsRhCG^R202C^-GFP did not reveal a reduced protein level compared to their respective native version (Fig. 2g and 2h). In keeping with the absence of deleterious phenotype related to the expression of the HsRhCG^T45A^ and HsRhCG^A347T^ variants, the corresponding protein levels were not reduced compared to native HsRhCG, the variants appearing even more produced than the native protein (Fig. S1).

Subcellular fractionation was further performed to test whether the mutations impair plasma membrane targeting of the functionally-altered Rh variants. We have previously shown that a fraction of heterologously expressed Rh proteins reaches the plasma membrane of yeast cells while a large part is retained in the endoplasmic reticulum [10]. Here, we found that the HsRhAG^I61R^-GFP, HsRhAG^F65S^-GFP and HsRhCG^R202C^-GFP proteins were not significantly impaired in reaching the plasma membrane of yeast cells compared to their native version (Fig. S2 and S3). Hence, the loss of function of the HsRhAG^I61R^, HsRhAG^F65S^ and HsRhCG^R202C^ variants is not due to major destabilization or misslocalization of the proteins. Taken together, these data show that the OHSt-associated I61R and F65S substitutions impair the inherent bidirectional ammonium transport activity of HsRhAG. These data also reveal that the HsRhCG^R202C^ variant, resulting from an nsSNP with unknown pathological outcome, is also impaired in its inherent bidirectional ammonium transport activity.

It has been proposed that unlike native HsRhAG, the I61R and F65S variants mediate the transport of potassium [27]. We have previously shown that expression of HsRhAG does not allow growth of a *trk1Δ trk2Δ* yeast mutant defective in endogenous potassium transporters [9]. While the F65S mutant behaved as native HsRhAG, the I61R variant conferred slow growth to *trk1Δ trk2Δ* cells under limiting potassium conditions (Fig. 2i). These data suggest that HsRhAG^I61R^ slightly alters potassium conductance in yeast.

### Analysis of potential trans-dominant effects of altered HsRhAG and HsRhCG variants

**Figure 3:**
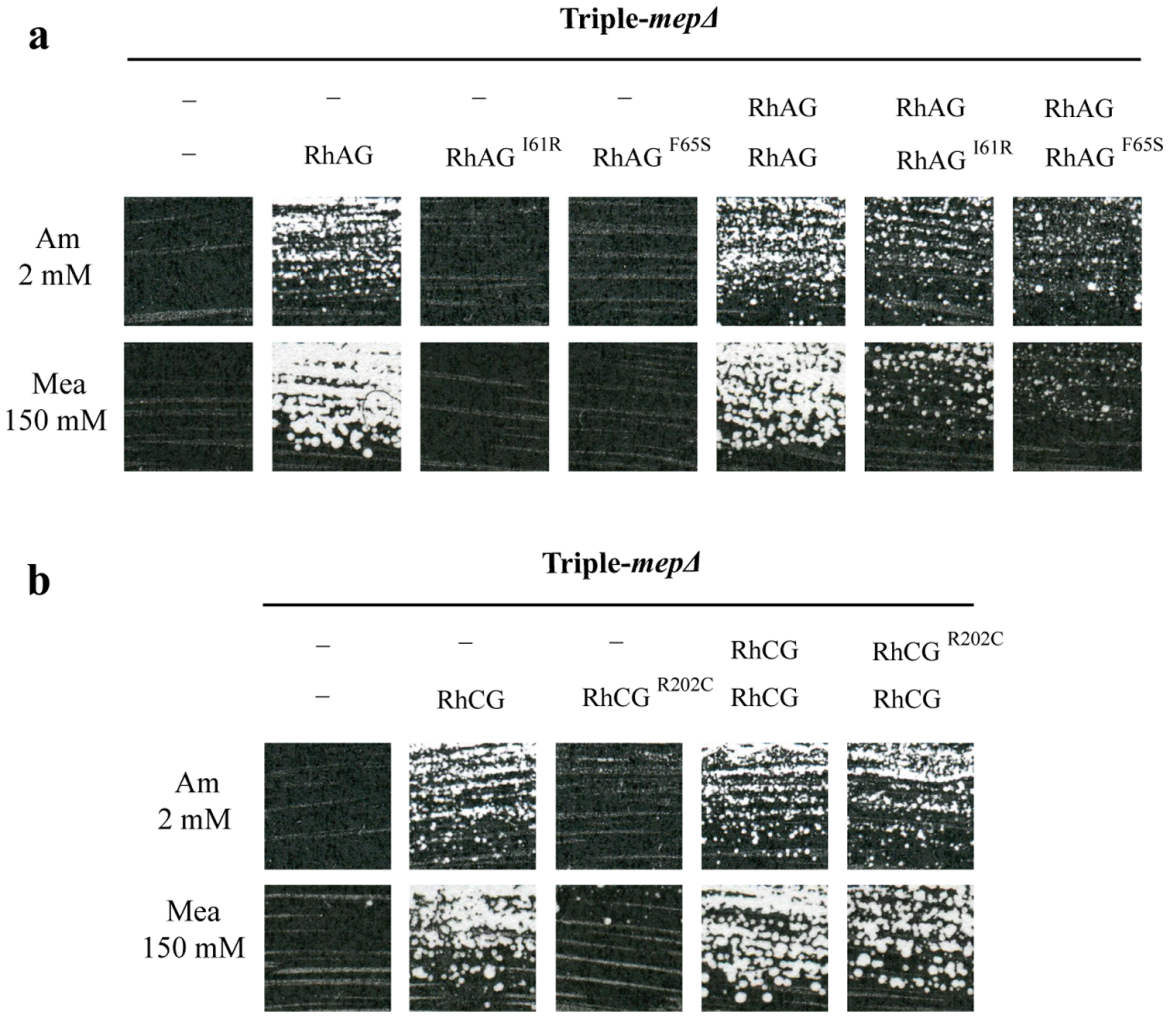
Analysis of trans-dominant effects of altered HsRhAG and HsRhCG variants. Growth tests on solid minimal medium containing as the sole nitrogen source 2 mM ammonium (Am 2 mM) or, proline supplemented with a toxic concentration of methylammonium (Mea 150 mM). **a** Triple-*mepΔ* cells (31064a) were co-transformed with 2 multi-copy vectors: p424 (−) or p424-HsRhAG with p426 (−), p426-sRhAG, p426-HsRhAG^I61R^ or p426-HsRhAG^F65S^. Cells were incubated for 7 days at 29°C. **b** Triple-*mepΔ* cells (31064a) were co-transformed with 2 multi-copy vectors: p424 (−), p424-HsRhCG or p424-HsRhCG^R202C^ with p426 (−), p426-HsRhCG or p426-HsRhCG^R202C^. Cells were incubated for 7 (Am 2 mM) and 8 (Mea 150 mM) days at 29°C.

Though HsRhAG monomers likely associate into multimeric complexes, it is yet unclear whether the OHSt-associated mutations in HsRhAG could have dominant negative impacts on the native proteins in heterozygous individuals. To test whether inactive HsRhAG and HsRhCG variants could induce trans-dominant effects on native counterparts, native and mutated Rh factors were co-expressed in triple-*mepΔ* cells from two independent multi-copy plasmids with different selection markers. When cells expressed HsRhAG^I61R^ or HsRhAG^F65S^ simultaneously with native HsRhAG, the growth efficiency was reduced compared to cells only expressing native HsRhAG (Fig. 3a). This was observed for both growth on low ammonium and resistance to methylammonium. On the other hand, co-expression of HsRhCG and HsRhCG^R202C^ had no clear impact on the growth of triple-*mepΔ* cells on low ammonium or on the resistance to methylammonium compared to cells only expressing native HsRhCG (Fig. 3b).

These results suggest that the OHSt-associated mutations in HsRhAG could partially alter the normal function of native HsRhAG. This could also be the case in heterozygous individuals affected by OHSt. In contrast, our data indicate that the HsRhCG^R202C^ does not alter the function of co-expressed native HsRhCG.

### *RHcg* gene-dose effect on urinary ammonium excretion in mice

Our data indicate that the HsRhCG^R202C^ variant, present in a part of the human population, has a strongly reduced intrinsic activity of bidirectional ammonium transport. Though HsRhCG^R202C^ does not appear to have trans-dominant negative effects on native HsRhCG, individuals bearing the R202C polymorphism at a heterozygous state might nevertheless have a limiting rate of ammonium transport with potential pathological outcomes. Using mice with two different backgrounds (CD1 and C57BL/6), we have previously shown that lack of MmRhcg is accompanied by a reduction in urinary ammonium excretion [25]. This defect was already detectable at baseline and was more pronounced under distinct acid challenges, i.e. upon 16h of fasting and after NH_4_Cl or HCl, acute and chronic, acid-load. Acid-loading of *Rhcg ^−/−^* mice provoked metabolic acidosis, with a sustained reduction of blood pH and bicarbonate level.

**Figure 4:**
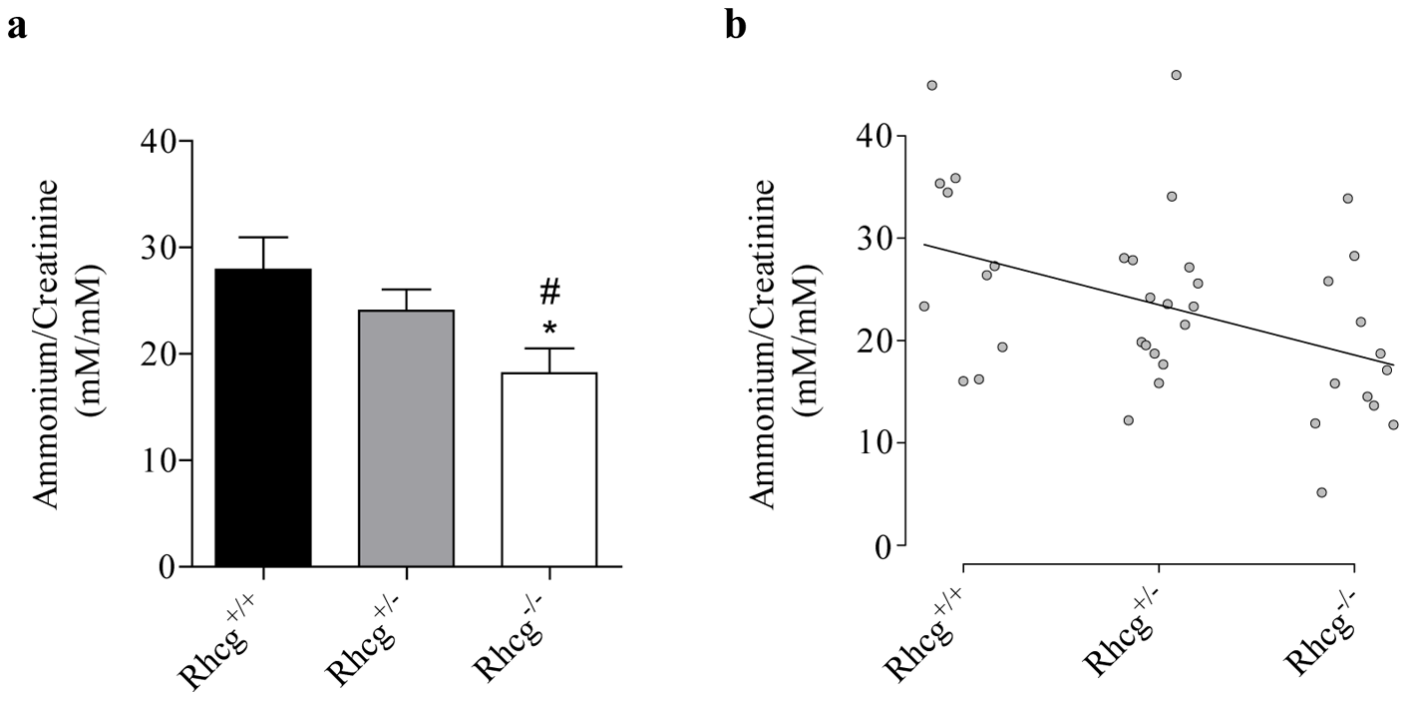
*RHcg* gene-dose effect on urinary ammonium content in mice. Urinary ammonium and creatinine were assayed on urines of *Rhcg^+/+^*, *Rhcg^+/−^* and *Rhcg^−/−^* mice collected after a fasting of 16 hours. **a** Data expressed as means ± s.e.m.; 10≤n≤16; *, p<0.05 versus *Rhcg^+/+^*; #, p<0.05 versus *Rhcg^+/−^*. **b** Correlation of Spearman. The correlation between *RHcg* alleles number (0, 1 or 2) and ammonium urinary content is positive (r_s_  = 0.425) and the gradation observed between *Rhcg^−/−^*, *Rhcg^+/−^* and *Rhcg^+/+^* is significant (p<0.05).

We used the mouse model to check whether a gene-dose effect in *Rhcg^+/−^* heterozygous mice could lead to a detectable effect on the urinary ammonium excretion as it occurs for *RHcg* knockout mice. Wild-type *Rhcg^+/+^*, heterozygous *Rhcg^+/−^* and homozygous *Rhcg^−/−^* mice were fasted for 16 hours to induce acidosis and parallel increase in urinary ammonium excretion, and the ammonium content of urine was determined. As expected *Rhcg^−/−^* showed a significant reduction in urinary ammonium content (Fig. 4a). *Rhcg^+/−^* mice displayed an intermediate urinary ammonium content compared to *Rhcg^+/+^* and *Rhcg^−/−^* mice. Moreover, significant correlation between the number of *RHcg* alleles and urinary ammonium content was observed, indicating a gene-dose effect (Fig. 4b). These data reveal that when mice are fasted for 16h, there is an *RHcg* gene-dose effect on urinary ammonium excretion. Hence, a reduced level of functional MmRhcg could be a limiting factor in this process.

### Arg211 of yeast ScMep2 equivalent to Arg202 of HsRhCG is similarly required for inherent ammonium transport activity

**Figure 5:**
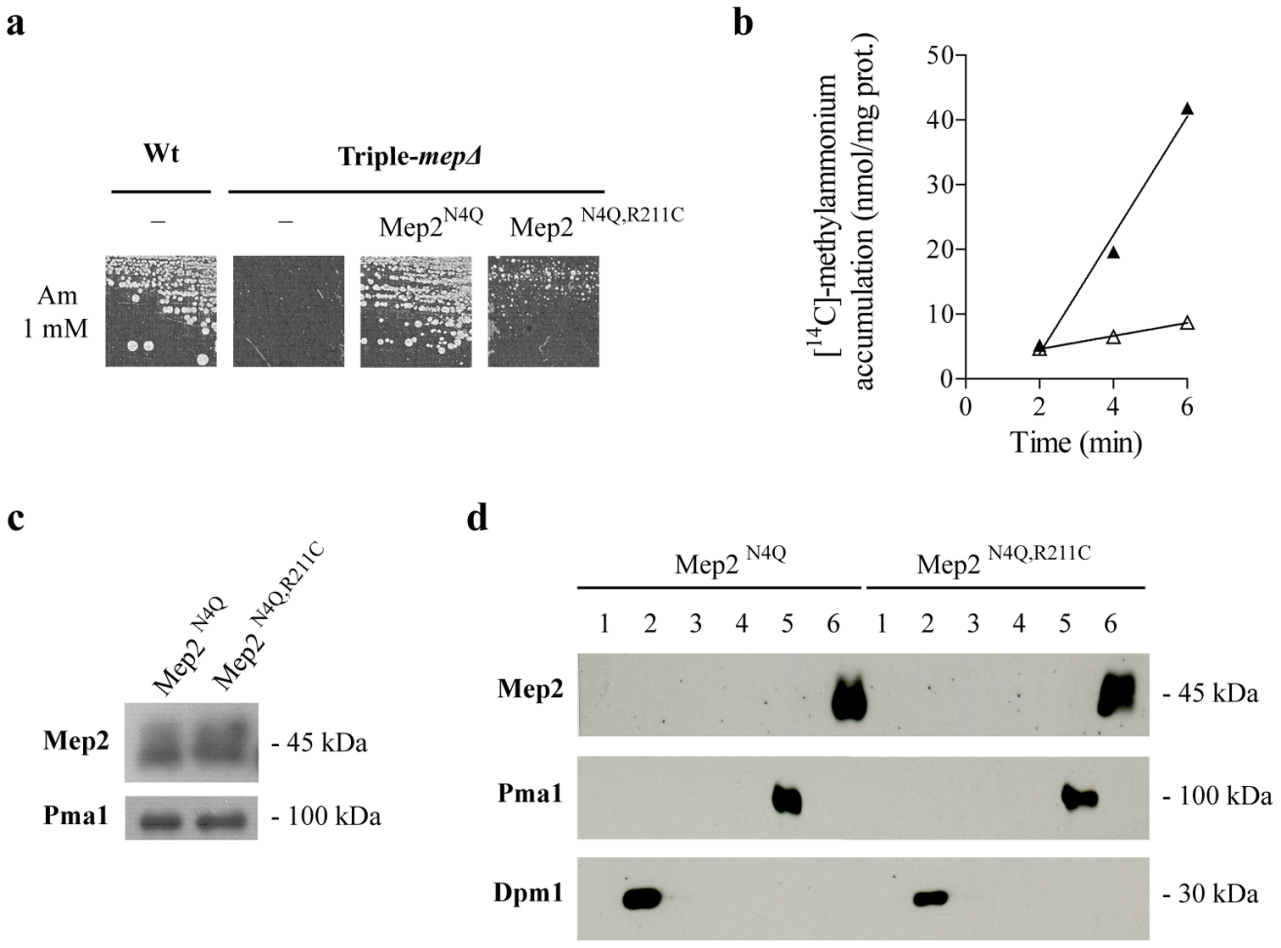
The R211C substitution alters inherent ammonium transport via yeast ScMep2. **a** Growth tests of yeast strains on solid minimal medium containing 1 mM ammonium (Am 1mM) as the sole nitrogen source. Wild-type cells (23344c) were transformed with the empty pFL38 vector (−) and triple-*mepΔ* cells (31019b) were transformed with the empty pFL38 vector or with YCpMep2^N4Q^ or YCpMep2^N4Q,R211C^. Cells were incubated for 4 days at 29°C. **b** Accumulation of [^14^C]-methylammonium (0.5 mM) was measured in proline-grown triple-*mepΔ* (31019b) cells transformed with YCpMep2^N4Q^ (▴) or with YCpMep2^N4Q,R211C^ (*Δ*) **c** Immunodetection of the ScMep2 variant. Same cells used in (b). Membrane-enriched cell extracts were separated by SDS-PAGE and immunoblotted with anti-Mep2 antibodies. ScPma1 was immunodetected as a loading control. **d** Subcellular localization of the ScMep2 variant. Same cells used in (b). Membrane-enriched yeast cell extracts were submitted to subcellular fractionation. The six different fractions were separated by SDS-PAGE and immunoblotted with anti-Mep2 antibodies. ScDpm1 and ScPma1 were immunodetected as markers for internal membranes (fractions 2, 3 and 4) and for plasma membrane (fractions 5 and 6), respectively.

Basic residues are usually found at the analogous position of HsRhCG Arg202 in Mep-Amt-Rh members and more particularly in Mep-Amt proteins (Fig. 1b). To test whether this basic residue is functionally relevant in Mep-Amt proteins as well, an arginine to cysteine substitution was introduced in yeast ScMep2 at position 211. We used site-directed mutagenesis on a plasmid allowing the expression of the ScMep2^N4Q^ protein of *S. cerevisiae*. Mep2^N4Q^ corresponds to the unglycosylable ScMep2 form which is readily detectable as discrete bands though conserving the major kinetic properties of native ScMep2 [32]. Growth tests showed that the ScMep2^N4Q,R211C^ has a strongly reduced ability to repair the growth defect of triple-*mepΔ* cells on low ammonium compared to ScMep2^N4Q^ (Fig. 5a). Prolonged incubation of the cells revealed that ScMep2^N4Q,R211C^ however conserves a residual activity, as was observed for HsRhCG^R202C^ (data not shown). Measurement of [^14^C]-methylammonium (0.5 mM) accumulation revealed that ScMep2^N4Q,R211C^ is affected in substrate transport efficiency (Fig. 5b). Moreover, western blot experiments showed that ScMep2^N4Q,R211C^ is not destabilized (Fig. 5c), and that the protein correctly reaches the plasma membrane (Fig. 5d).

Hence, yeast ScMep2^N4Q,R211C^, like human HsRhCG^R202C^, is also affected in its inherent ammonium transport activity. These data reveal that the conserved arginine residue is important to sustain a high rate of substrate transport in both proteins.

### The C-terminal extension of HsRhCG forms an inter-subunit network with intracellular loops

**Figure 6:**
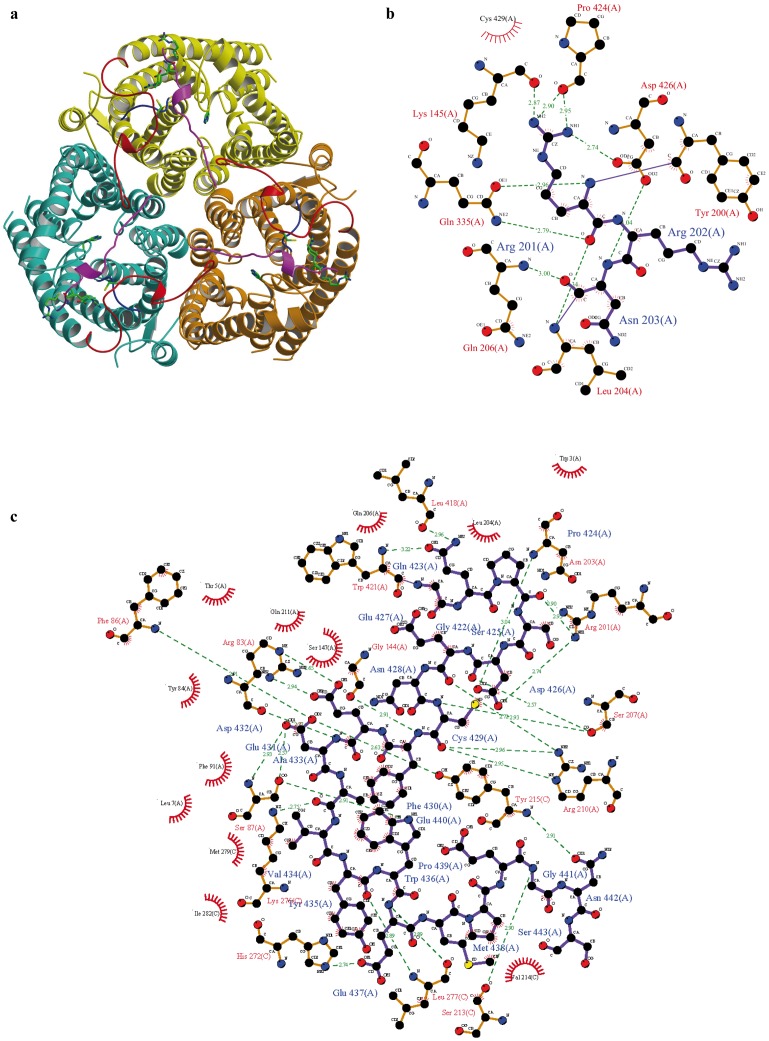
The C-terminal extension of HsRhCG forms a network with intracellular loops. **a** A cytoplasmic view of the trimeric HsRhCG crystal structure. Each monomer is depicted in coloured ribbon. To emphasize contacts between distant residues, the long intracellular loop between transmembrane helices TM5 and TM6 is in magenta, the short loop between TM3 and TM4 in blue and the C-terminus extension from Arg417 to Ser443 is coloured in red. The C-extension of one monomer is found oriented towards the adjacent monomer, in a circular manner. Note that the last C-terminal 36 residues of HsRhCG protein cannot be built due to lack of available structural data. In order to localize the substrate pore channel, side chains of the twin-histidines (His185 and His344) are showed and labelled in each monomer. The side chain of Arg202, as well as those of the two surrounding residues, Arg201 and Asn203, are also depicted and labelled in each monomer. The atomic colour scheme is carbon in green, nitrogen in blue, oxygen in red and sulphur in yellow. **b** Schematic 2D interacting plot of residues Arg201 to Asn203 in HsRhCG crystal structure. Hydrogen bonds are green dashed lines and van der Waals contacts are represented by red semi-circles with radiating spokes. Bonds of Arg202 and the two surrounding residues Arg201 and Asn203 are in purple and other bonds are in light brown. All atoms and residues are labelled. The atomic colour scheme is carbon in dark, nitrogen in blue, oxygen in red and sulphur in yellow. **c** Schematic 2D interacting plot of residues 422 to 443 of the HsRhCG C-terminus. See (b) for the legend.

We examined the crystal structure of HsRhCG (pdb code 3HD6) to localise Arg202 and visualize neighbouring residues (Fig. 6a). The side chain of the arginine is oriented towards the solvent and appears not involved in interactions. However, the Arg201 and Asn203 residues surrounding Arg202 take part in an intense network of interactions implicating the cytosolic C-terminal extension of HsRhCG (Fig. 6b and 6c). Arg201 forms a salt bridge with Asp426, and Asn203 is hydrogen bonded to Asp426. Side-chain/side-chain contacts are also observed between Arg201 and Cys429. Gly144 and Ser147 in the TM3-TM4 intracellular loop are in proximity of the same region of the C-terminus. Residues of the HsRhCG C-terminus further contact loops of the contiguous monomer in the trimeric association (Fig. 6c). These data suggest that the loss of transport function in HsRhCG^R202C^ could be consequent to the alteration of the interactions linking intracellular loops to the cytosolic C-terminus.

## Discussion

### Pathological SNPs alter the inherent bidirectional ammonium transport activity of HsRhAG

We have characterized the two HsRhAG polymorphisms so far associated to OHSt and we show that both I61R and F65S variants display a loss-of-function in their inherent activity of bidirectional ammonium transport. This is consistent with data showing a reduced [^14^C]-methylammonium content measured in xenopus oocytes expressing the F65S variant [28] and with the reduced ammonia-induced alkalinisation of OHSt red cells expressing the same variant [29]. A stomatocytosis cation leak has been associated to several missense mutations, not only in HsRhAG, but also in the bicarbonate/Cl^−^ exchanger AE1 and the glucose/ascorbate transporter GLUT1 [28], [33], [34]. It remains unclear whether the cation leak is directly and solely linked to a modified conductance of these mutated proteins and/or to a subsequent activation of common cation transport pathways in stomatocytosis red cells. If the latter is true, the pathways remain to be identified. Both I61R and F65S mutations were initially proposed to confer monovalent cation conductance to HsRhAG [27]. Bruce and collaborators proposed that in the particular case of the F65S variant, there would be a widening of the pore constriction to dimensions exceeding the hydrated ionic radii of Na^+^, K^+^, and NH_4_^+^. However, the modelling used did not take into account the dynamics and the local fluctuations of the protein structure. Moreover, smaller residues like Ile and Val are naturally found in several members of the Mep-Amt-Rh family. That residue alteration at this position changes the pore radius with consequences for cation conductance appears not straightforward and would require more investigation.

The cation leak appears, to some extent, to be reproduced upon heterologous expression of the human HsRhAG variants in xenopus oocytes [28]. Of note, the leak was not only induced by the F65S variant but also by expression of native HsRhAG. Activities of endogenous Na^+^,K^+^-ATPase and Na^+^-K^+^-2Cl*^−^* cotransporter were found increased, likely as a secondary mechanism compensating for the leak [28].

In this context, we tested the ability of HsRhAG variants to transport K^+^ in yeast cells deprived of endogenous Trk potassium transporters. The two missense mutations produced slightly different phenotypes, with only the I61R variant allowing slow growth at limiting potassium concentrations. The F65S mutation has been reported so far in 6 families and 4 patients while the I61R variant is found in one single individual [27], [28]. The two mutations were associated to distinct sub-types of OHSt on the basis of phenotypic difference, including defective *in vitro* process of ATP-dependent endocytic vesiculation, which is found in normal red cells [35]. Our data also suggest a difference in both variants, in their impact on K^+^ conductance in yeast.

HsRhAG proteins associate into multimeric complexes [36]. Our data suggest that HsRhAG^I61R^ and HsRhAG^F65S^ are able to interact with native HsRhAG when co-expressed in yeast cells and reduce the ammonium transport activity of the heteromers. A trans-dominant negative effect could also occur in heterozygous OHSt individuals. Moreover, in red cells, HsRhAG has been shown to be part of the AE1 (Band3) macrocomplex that links several membrane proteins to the cytoskeleton [37]. In this complex, HsRhAG directly interacts with AE1. Glut1 might also be part of this macrocomplex as it was shown to interact physically with AE1 [38]. It is conceivable that the stomatocytosis mutations in AE1, Glut1 or HsRhAG alter the integrity of this macrocomplex. The mutated variants might titrate or liberate a component of the complex associated to the membrane or to the cytoskeleton which could then play a secondary role on the activation of cation transport pathways responsible for the stomatocytosis cation leak. Metabolomics studies were recently performed on red cells of 4 patients expressing the HsRhAG^F65S^ variant [39]. A raise in the concentrations of glycolysis end-products, such as pyruvate or lactate, were found as a metabolic signature of OHSt patients' erythrocytes. Increased glycolysis was proposed to provide the high levels of ATP required for the raised Na^+^/K^+^ ATPase activity likely compensating for the cation leakage.

Ammonium has been shown to be 3 times more concentrated in erythrocytes compared to plasma [40]. As our observations reveal that HsRhAG^I61R^ and HsRhAG^F65S^ display a loss of ammonium transport function, it is also conceivable that stomatocytosis could directly result from an alteration of the ammonium content of these cells.

### Identification and characterization of a potentially pathological SNP in HsRhCG

Absence of mouse Rhcg is associated to pH alteration of body fluids and to anomalies characteristic of incomplete forms of a human syndrome termed “distal renal tubular acidosis” (dRTA) [25]. The critical role of renal acid elimination is underscored by a variety of syndromes of acquired or inherited forms of renal tubular acidosis, chronic metabolic acidosis representing a main morbidity and mortality risk factor. Chronic metabolic acidosis may even accelerate deterioration of renal function in patients with early stages of renal disease [41]. The frequency of chronic metabolic acidosis is expected to increase with the anticipated rise in chronic kidney diseases in our aging population.

Hereditary forms of dRTA have been associated to mutations in the H^+^ V-ATPase and in the AE1 bicarnonate/Cl*^−^* exchanger [26]. Though HsRhCG is a candidate gene for hereditary forms of dRTA, no associated mutations have been uncovered so far. Here we show that the nsSNP leading to the R202C substitution in human HsRhCG is accompanied by an altered efficiency in inherent bidirectional ammonium transport. Ammonium constitutes the most important urinary buffer. The measurement of urinary ammonium excretion allows assessing renal acidification. In mice, two Rhesus proteins appear to contribute to ammonium elimination in the collecting duct. In addition to MmRhcg, one study reports that basolateral Rhbg also contributes to the process [42]. MmRhcg appears however to be rate-limiting as our observations reveal an *RHcg* gene-dose effect on urinary ammonium excretion. This effect is already detectable after 16h of fasting but might be even more important during chronic acidosis. The minor allele frequency of the nsSNP leading to the R202C substitution in HsRhCG is reported to be of about 9%. Compared to Asian, Sub-Saharan African and African American populations, the European population shows the highest frequency of the minor allele and also comprises individuals homozygous for this mutation. A reduction of functional HsRhCG proteins in individuals showing this nsSNP might underlie disorders leading to metabolic acidosis, but might also alter the processes of ammonium detoxification.

### The C-terminal extension of HsRhCG could be involved in a conserved mechanism of gating control

Despite the increasing number of functional characterizations and of 3D views provided by the available structures, the nature of the substrate and the transport mechanism of Mep-Amt-Rh proteins remain a matter of debate. Electrogenic transport of NH_4_^+^ (or NH_3_ + H^+^) and electroneutral transport of NH_3_ (or NH_4_^+^/H^+^ antiport) are both reported in both Mep-Amt and Rh subfamilies [11], [15], [16], [43]–[46]. It is conceivable that proteins with distinct transport mechanisms co-exist in each Mep-Amt and Rh subfamilies. Cl*^−^* channels and Cl*^−^*/H^+^ antiporters were shown to share a similar architecture though having a different transport mechanism [47]–[49]. In this line, we have shown that important functional differences exist between yeast ScMep2 and ScMep1; a single point mutation in ScMep2 allowing the protein to mimic the transport properties of Mep1 [50].

3D views of both Mep-Amt and Rh proteins reveal the presence of a gate formed by two stacked phenylalanines side chains hindering the access to the pore [11]–[16]. Structural data of a bacterial Mep-Amt show that the portion of the C-terminal extension, conserved among all Mep-Amts, interacts with intracellular loops of its own monomer and also with the loops of the contiguous one in a circular manner [13]. These interactions were proposed to participate in a gating process of the *Arabidopsis thaliana* Amt1;1 protein [20], [21].

A similar gating mechanism could also exist in Rh proteins though the C-terminal extension is divergent (Fig S4). Analysis of the structure of trimeric HsRhCG indicates that the C-terminal extension of each subunit also contacts intracellular loops in a circular manner. The R202C polymorphism in HsRhCG and the analogous R211C mutation in yeast ScMep2 both lead to proteins with a low basal activity. It is tempting to propose that the R202C could alter a gating process tuned by the HsRhCG C-terminus.

The divergence of the C-terminus between Mep-Amt and Rh proteins could reflect an adaptation to the sequence divergence also noticed in the respective intracellular loops. These differences could also allow the fine-tuning of interactions with potential regulators to respond to specific physiological variations though preserving a similar gating mechanism. For instance, such a gating control is mediated by GlnK proteins on Mep-Amts in bacteria [51]. However, GlnKs are not conserved in eukaryotes, except in some plants. It should be noted that the last 42 C-terminal residues are lost in the electronic density of the HsRhCG crystal structure revealing the high flexibility of this region. This extension could possibly adopt a defined structure by interacting with a particular protein partner, and play a regulatory role.

Membrane proteins constitute more than 30% of the proteins encoded by the human genome. A still growing number of diseases are linked to defects in transport systems and more than 60% of therapeutic targets are membrane proteins [52]–[54]. Nowadays central questions in genetic studies are to identify functional DNA variants having clinical impacts on a disease or phenotype of interest. An increasing number of SNPs are being reported for Rh proteins. The dbSNP database lists up to 606 SNPs in the *RHCG* gene, including synonymous, non synonymous and frameshift SNPs, in intronic and exonic regions of the gene. 42 of the SNPs are non synonymous, leading to a change in amino acid sequence. However, there is currently very little information on the functional consequences of these variants. Rhesus proteins are part of the few human transport systems that conserve their functionality upon heterologous expression in yeast [9], [10]. Yeast could be used as a potent screening and phenotyping tool to characterize additional Rh SNPs and estimate the functionality of the resulting Rh variants in isolation from any animal context and thus from any other variation of the human genome.

Based on the results of this study, we would predict that the R202C variant of HsRhCG would have decreased transport activity in the kidney leading to decreased ammonium elimination and impaired acid elimination. Though the potential clinical impact of this missense mutation remains to be evaluated, it may be associated to an increased susceptibility to develop metabolic acidosis and associated disorders.

## Materials and Methods

### Ethics statement

Animal care and experimental procedures were approved by the local ethics committee CEBEA (Comité d'Ethique et de Bien-Etre Animal, Institut de Biologie Moléculaire et Médicale, Université Libre de Bruxelles, Gosselies, Belgium). The mice used in this study were housed under specific pathogen-free conditions in our animal facility, laboratory license LA 1500474.

### Yeast strains, growth conditions, methods

*S. cerevisiae* strains 23344c, 31019b and 31064a are isogenic with the wild-type strain Σ1278b [55], as is CY162 [56] with the wild-type strain S288c [57], except for the mutations mentioned (Table S1). Cells were grown in a minimal buffered (pH 6.1) medium with 3% glucose as the carbon source [58]. To this medium, nitrogen sources were added as required by the experiment. The nitrogen sources used were 0.1% glutamate, 0.1% proline, 0.1% arginine or (NH_4_)_2_SO_4_ at the specified concentration. Methylammonium was used at 150 mM. Methionine (1 mM) was added to the medium to repress the expression of *RHCG* or *RHAG* genes [59]. For growth tests on limiting potassium concentrations, a Yeast Nitrogen Base medium (MP Biomedicals) deprived of potassium salts was used and was supplemented with 3% glucose as the carbon source, 0.1% glutamine as the sole nitrogen source, NaH_2_PO_4_ (7.35 mM) as the phosphate source and KCl was added at a concentration of 5 mM. Yeast cells were transformed as described [60]. Plasmids used in this study are listed in Table S1. Site-directed mutageneses of *RHCG, RHAG* and *MEP2* were performed using the Quick Change Site-directed Mutagenesis Kit (Stratagene). Primers used are available on request. Correct mutagenesis was checked by sequencing.

### Western immunoblotting

Membrane-enriched cell extracts and western blotting were performed as described previously [61]. ScMep2 protein was probed with a rabbit antiserum (1∶1000) raised against the C-terminal region of the proteins [32]. HsRhCG was probed with an anti-HsRhCG antibody (1∶1000; SantaCruz). HsRhAG and HsRhCG proteins tagged to GFP were probed with anti-GFP (1∶5000) antibody, and ScPma1 and ScDpm1 proteins with anti-Pma1 (1∶10000) [62] and anti-Dpm1 (1∶5000) (Molecular Probes) antibodies. Primary antibodies were detected with horseradish-peroxidase-conjugated anti-rabbit and anti-mouse-IgG secondary antibodies followed by measurement of chemoluminescence (Lumi-Light^PLUS^, Roche).

### Subcellular Fractionation

Fractionation was performed essentially as described previously [50]. About 10^8^ yeast cells were filtered (Millipore 0.45 µm), washed with cold water, and resuspended in 0.2 ml buffer L (25 mM Tris-HCl pH 8, 2.5 mM EDTA) containing proteases inhibitors (Complete-Mini, Roche). An equal volume of glass beads was added and cells were lysed at 4°C by vortex mixing for 10 minutes. The extracts were diluted with 500 µl buffer L and centrifuged for 5 min at 3000 rpm to remove unbroken cells and large debris. The cleared lysate was centrifuged at 14500 rpm for 20 min in a SW55 Ti rotor (Beckman). The pellet (P20) was then resuspended in 20% glycerol in buffer B (10 mM Tris-HCl pH 7.4, 0.2 mM EDTA, 0.2 mM DTT). The samples (500 µl) were loaded onto the top of a sucrose step gradient (0.5 ml 53%, 1 ml 43%, in buffer B) and centrifuged at 33000 rpm for 2 h in a SW55 Ti rotor (Beckman). After centrifugation, six fractions of equal volume were collected from the top of the gradient, and the distribution of HsRhCG-GFP, HsRhAG-GFP, ScMep2, Pma1 and Dpm1 was analysed by western blotting.

### [^14^C]-Methylammonium uptake assays

[^14^C]-methylammonium (0.5 mM) accumulation was measured as previously described [31], [63] with cells grown in minimal medium containing proline as the nitrogen source. Briefly, 5-ml samples of an exponentially growing culture corresponding to about 0.25 mg of protein per ml were put, without any change of the medium, into vessels containing the labelled methylammonium and preheated to 29°C in a rotary water bath. 1-ml samples were then removed at time intervals and poured onto filters (Millipore 0.45 µm) which were immediately washed with 5 times 2-ml iced water before counting.

### Ammonium removal assays

Ammonium removal assays were performed as previously described [31]. Briefly, triple-*mepΔ* cells (31019b) transformed with an empty vector or with a vector allowing *RH* expression under the control of the MET25 promoter were first grown exponentially on proline medium (pH 6.1) containing 1 mM methionine to repress the *RH* gene and then transferred to fresh proline medium without methionine to induce *RH* gene expression. After 180 min of induction, equivalent amounts of cells were transferred to fresh proline medium buffered at pH 6.1, ammonium (2 mM) was added, and samples of the medium were withdrawn at time intervals to follow ammonium removal. The ammonium content in medium samples was determined by coupling with L-glutamate dehydrogenase.

### Ammonium excretion assays

Ammonium excretion assays were performed as previously described [9]. Triple-*mepΔ* cells (31019b) transformed with an empty vector or with a vector allowing *RH* expression under the control of the MET25 promoter were first grown exponentially on arginine (0.1%) medium (pH 6.1) containing 1 mM methionine to repress *RH* gene. Cells were then transferred to arginine medium without methionine to induce *RH* gene expression, samples of supernatant were withdrawn at time intervals, and their ammonium concentration assayed by coupling with L-glutamate dehydrogenase.

### Urinary ammonium excretion assays

Experiments were performed on adult (3–4 months old) male *Rhcg^+/+^*, *Rhcg^+/−^* and *Rhcg^−/−^* CD1 mice [25]. “One spot” urines were collected after a fasting of 16 hours with water *ad libitum*. Ammonium concentrations in the urine samples were measured by L-glutamate deshydrogenase determination. Ammonium concentrations are reported to urinary creatinine levels. The creatininuria was assayed with the CR01 kit (Oxford Biomedical Research).

### Statistics

Data are expressed as means ± s.e.m. Statistical comparisons were assessed by Student's *t*-tests using SigmaPlot software. The relationship between the *RHcg* alleles number versus urinary ammonium content was assessed using the Spearman rank correlation coefficient (*r*_s_). Differences were considered significant when the probability is below 0.05 (p<0.05).

### Sequence and structural analyses

For multiple alignments, sequences were retrieved from the NCBI or UniProtKB/SWISS-PROT databases, aligned with clustalW and edited with GeneDoc. Bioedit program was also used for visual inspection and graphical generation of sequence alignments. The molecular graphics softwares, PyMol [64] and Coot [65] were used for visualization and interaction analysis. Schematic 2D interacting plots were generated using the program LigPlot [66]. The Figure 6a was produced using successively MolScript [67] and Raster3D [68] programs.

## Supporting Information

Figure S1 Immunodetection of the T45A and A387T variants of HsRhCG expressed in yeast. Triple-*mepΔ* cells (31019b) transformed with the empty p426 vector (−) or a multi-copy plasmid bearing *HsRHCG*, *HsRHCG^T45A^* or *HsRHCG^A387T^* were grown on glutamate minimal medium. Membrane-enriched cell extracts were separated by SDS-PAGE and immunoblotted with anti-RhCG antibodies (SantaCruz). ScPma1 was immunodetected as a loading control.
(TIF)

Figure S2 a Subcellular localization of OHSt-related variants. Triple-*mepΔ* cells (31019b) transformed with the empty p426 vector (−), or with a multi-copy plasmid bearing GFP-tagged native (HsRhAG-GFP) or mutated *HsRHAG* genes (HsRhAG^I61R^-GFP, HsRhAG^F65S^-GFP) were grown on glutamate minimal medium. Membrane-enriched yeast cell extracts were submitted to subcellular fractionation. The six different fractions were separated by SDS-PAGE and immunoblotted with anti-GFP antibodies. ScDpm1 and ScPma1 were immunodetected as markers respectively for internal membranes (fractions 2, 3 and 4) and for plasma membrane (fractions 5 and 6). **b** Quantification (%) of the proportion of HsRhAG-GFP variants reaching the plasma membrane of the yeast. The graphic, based on the quantification of the immunoblot, represents the proportion of proteins in fractions 5 and 6 compared with total HsRhAG-GFP proteins.
(TIF)

Figure S3 a Subcellular localization of HsRhCG^R202C^ variant. Triple-*mepΔ* cells (31019b) transformed with the empty p426 vector (−), or with a multi-copy plasmid bearing *HsRHCG-GFP* or *HsRHCG^R202C^-GFP* were grown on glutamate minimal medium. Same as in Fig. S2a. **b** Quantification (%) of the proportion of HsRhCG^R202C^-GFP variant reaching the plasma membrane of the yeast. Same as in Fig. S2b.
(TIF)

Figure S4 Primary sequence alignment of the last transmembrane domain (TM11) and adjacent part of the C-terminus of Mep-Amt-Rh proteins from different organisms. a All the selected Mep-Amt-Rh sequences were first aligned using clustalW. No apparent C-terminal amino acid conservation is revealed considering the complete alignment. **b–c** Analysis of alignments focused on each Mep-Amt or Rh subfamily reveals subfamily conservation within the C-terminus. **a–c** SWISS PROT or GeneBank accession numbers are referred hereafter. ScMep1, 2, 3: *S. cerevisiae* Mep1 (P40260), Mep2 (P41948) and Mep3 (P53390); CaMep1, 2: *C. albicans* Mep1 (Q5AJH9) and Mep2 (Q59UP8); EcAmtB: *E. coli* AmtB (P69681); AfAmt1, 2, 3: *A. fulgidus* Amt1 (O29285), Amt2 (O28528) and Amt3 (O28525); AtAmt1_1, 1_2, 1_3, 1_4, 1_5, 2: *A. thaliana* Amt1;1 (P54144), Amt1;2 (Q9ZPJ8), Amt1;3 (Q9SQH9), Amt1;4 (Q9SVT8), Amt1;5 (Q9LK16) and Amt2 (Q9M6N7); NeRh: *N. europaea* Rh50 (Q82X47); GcRh: *G. cydonium* Rh (O18432); CeRh1, 2: *C. elegans* Rh1 (Q22947) and Rh2 (Q17463); CrRh1, 2: *C. reinhardtii* Rh1 (Q94CJ2) and Rh2 (Q8RUE9); DmRh: *D. melanogaster* Rh (Q9V3T3); MmRhag, bg, cg, d: *M. musculus* Rhag (Q9QUT0), Rhbg (Q8BUX5), Rhcg (Q9QXP0) and Rhd (Q8CF94); HsRhAG, BG, CG, D, CE: *H. sapiens* RhAG (Q02094), RhBG (AAL05978), RhCG (Q9UBD6), RhD (Q02161) and RhCE (P18577).
(TIF)

Table S1 List of yeast strains and plasmids used in this study.
(DOC)
