# Supplementary figures and images for: SNPs Altering Ammonium Transport Activity of Human Rhesus Factors Characterized by a Yeast-Based Functional Assay
Source: PLoS One. 2013 Aug 13;8(8):e71092. doi: 10.1371/journal.pone.0071092 (PMC3742762; doi:10.1371/journal.pone.0071092)

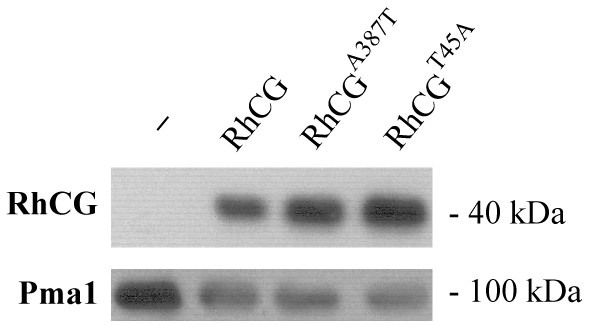

Supplement: Figure S1 — Immunodetection of the T45A and A387T variants of HsRhCG expressed in yeast. Triple-mepΔ cells (31019b) transformed with the empty p426 vector (−) or a multi-copy plasmid bearing HsRHCG, HsRHCGT45A or HsRHCGA387T were grown on glutamate minimal medium. Membrane-enriched cell extracts were separated by SDS-PAGE and immunoblotted with anti-RhCG antibodies (SantaCruz). ScPma1 was immunodetected as a loading control. (TIF) [file pone.0071092.s001.tif]

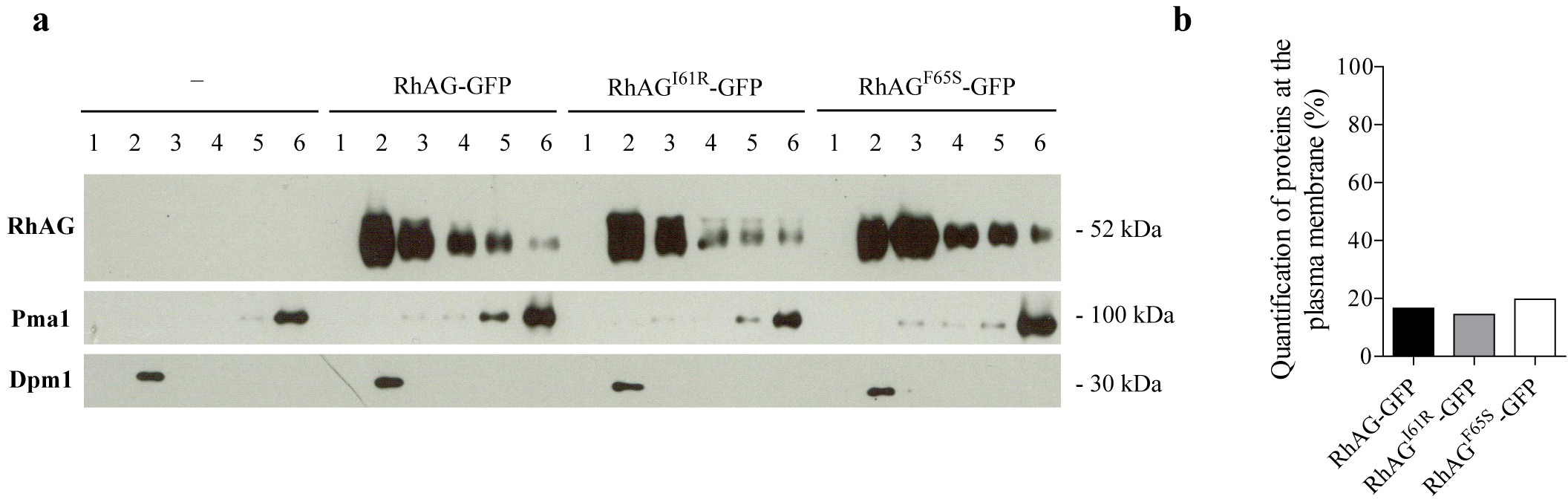

Supplement: Figure S2 — a Subcellular localization of OHSt-related variants. Triple-mepΔ cells (31019b) transformed with the empty p426 vector (−), or with a multi-copy plasmid bearing GFP-tagged native (HsRhAG-GFP) or mutated HsRHAG genes (HsRhAGI61R-GFP, HsRhAGF65S-GFP) were grown on glutamate minimal medium. Membrane-enriched yeast cell extracts were submitted to subcellular fractionation. The six different fractions were separated by SDS-PAGE and immunoblotted with anti-GFP antibodies. ScDpm1 and ScPma1 were immunodetected as markers respectively for internal membranes (fractions 2, 3 and 4) and for plasma membrane (fractions 5 and 6). b Quantification (%) of the proportion of HsRhAG-GFP variants reaching the plasma membrane of the yeast. The graphic, based on the quantification of the immunoblot, represents the proportion of proteins in fractions 5 and 6 compared with total HsRhAG-GFP proteins. (TIF) [file pone.0071092.s002.tif]

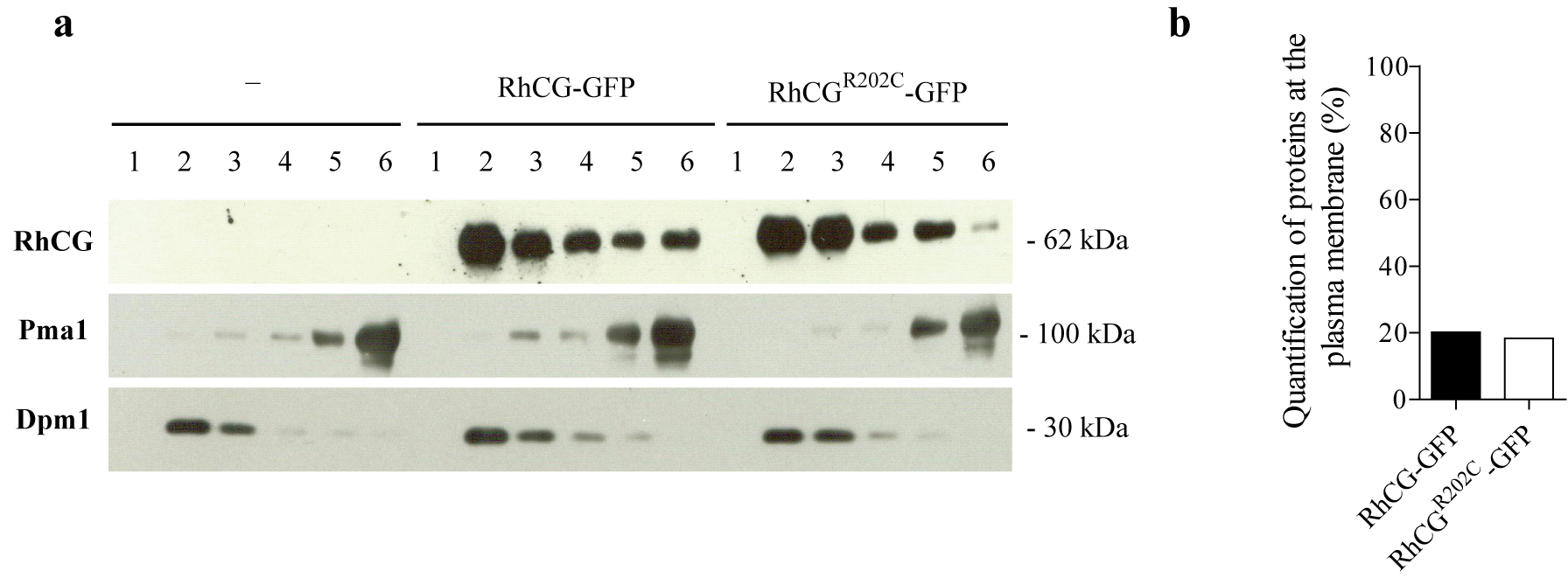

Supplement: Figure S3 — a Subcellular localization of HsRhCGR202C variant. Triple-mepΔ cells (31019b) transformed with the empty p426 vector (−), or with a multi-copy plasmid bearing HsRHCG-GFP or HsRHCGR202C-GFP were grown on glutamate minimal medium. Same as in Fig. S2a. b Quantification (%) of the proportion of HsRhCGR202C-GFP variant reaching the plasma membrane of the yeast. Same as in Fig. S2b. (TIF) [file pone.0071092.s003.tif]

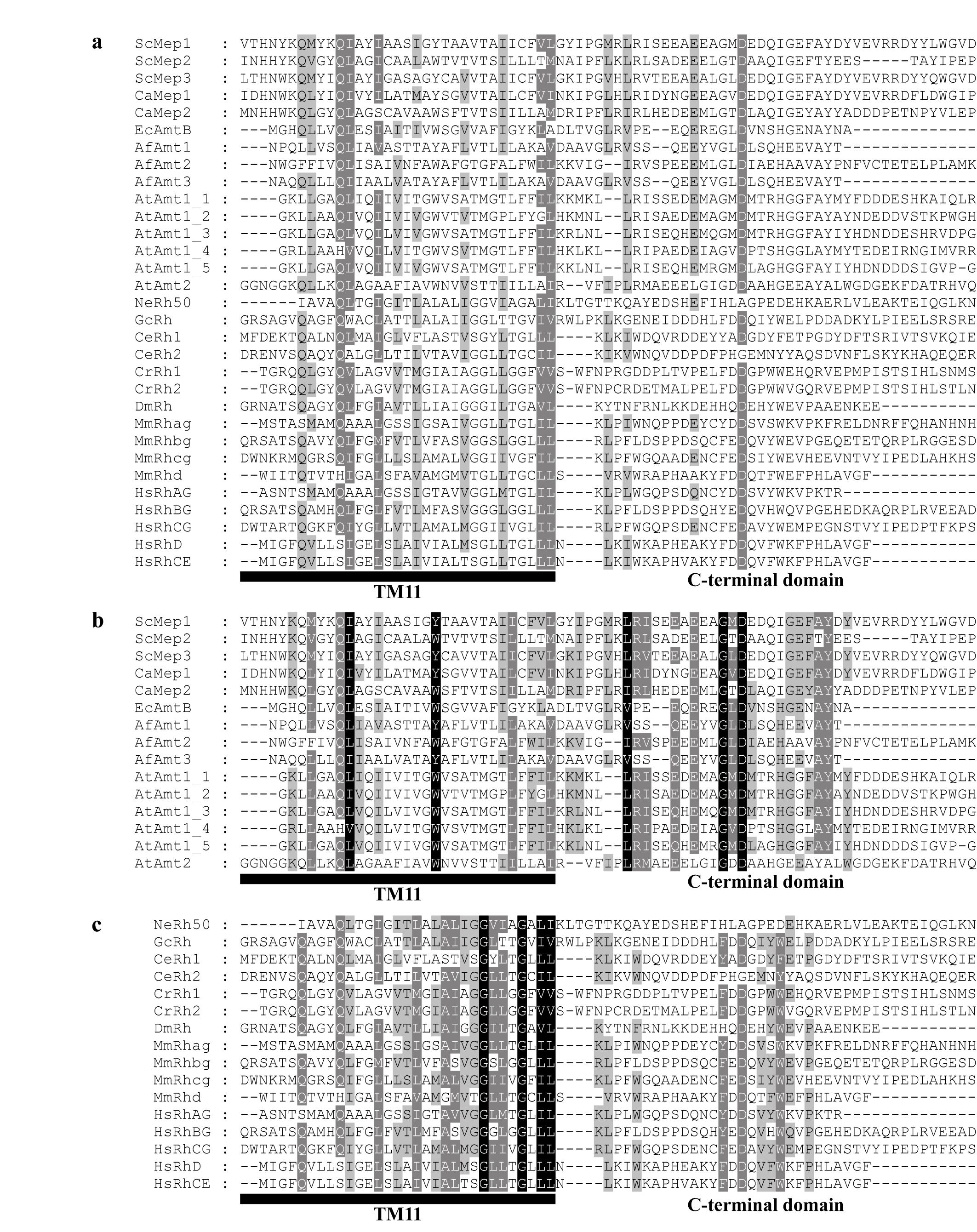

Supplement: Figure S4 — Primary sequence alignment of the last transmembrane domain (TM11) and adjacent part of the C-terminus of Mep-Amt-Rh proteins from different organisms. a All the selected Mep-Amt-Rh sequences were first aligned using clustalW. No apparent C-terminal amino acid conservation is revealed considering the complete alignment. b–c Analysis of alignments focused on each Mep-Amt or Rh subfamily reveals subfamily conservation within the C-terminus. a–c SWISS PROT or GeneBank accession numbers are referred hereafter. ScMep1, 2, 3: S. cerevisiae Mep1 (P40260), Mep2 (P41948) and Mep3 (P53390); CaMep1, 2: C. albicans Mep1 (Q5AJH9) and Mep2 (Q59UP8); EcAmtB: E. coli AmtB (P69681); AfAmt1, 2, 3: A. fulgidus Amt1 (O29285), Amt2 (O28528) and Amt3 (O28525); AtAmt1_1, 1_2, 1_3, 1_4, 1_5, 2: A. thaliana Amt1;1 (P54144), Amt1;2 (Q9ZPJ8), Amt1;3 (Q9SQH9), Amt1;4 (Q9SVT8), Amt1;5 (Q9LK16) and Amt2 (Q9M6N7); NeRh: N. europaea Rh50 (Q82X47); GcRh: G. cydonium Rh (O18432); CeRh1, 2: C. elegans Rh1 (Q22947) and Rh2 (Q17463); CrRh1, 2: C. reinhardtii Rh1 (Q94CJ2) and Rh2 (Q8RUE9); DmRh: D. melanogaster Rh (Q9V3T3); MmRhag, bg, cg, d: M. musculus Rhag (Q9QUT0), Rhbg (Q8BUX5), Rhcg (Q9QXP0) and Rhd (Q8CF94); HsRhAG, BG, CG, D, CE: H. sapiens RhAG (Q02094), RhBG (AAL05978), RhCG (Q9UBD6), RhD (Q02161) and RhCE (P18577). (TIF) [file pone.0071092.s004.tif]
