# Supplementary material for: SNPs Altering Ammonium Transport Activity of Human Rhesus Factors Characterized by a Yeast-Based Functional Assay
Source: PLoS One. 2013 Aug 13;8(8):e71092. doi: 10.1371/journal.pone.0071092 (PMC3742762; doi:10.1371/journal.pone.0071092)
Supplement: Table S1 — List of yeast strains and plasmids used in this study. (DOC) [file pone.0071092.s005.doc]

**Table S1.** List of yeast strains and plasmids used in this study.

| Strain or plasmid | **Genotype or description** | **Source or reference** |  |
| --- | --- | --- | --- |
|  |  |  |  |
| Strain |  |  |  |
| 23344c | *ura3* | Grenson, unpublished, lab collection | |
| 31019b | *mep1Δ mep2Δ::LEU2 mep3Δ::KanMX2 ura3* | [1] |  |
| 31064a | *mep1Δ mep2Δ::LEU2 mep3Δ::KanMX2 ura3 trp1* | This study |  |
| S288c |  | [2] |  |
| CY162 | *trk1Δ trk2Δ ura3 his3 his4* | [3] |  |
|  |  |  |  |
| **Plasmid** |  |  |  |
| p426 | pRS426MET25 *URA3* | [4] |  |
| p426-HsRhCG | p426MET25 *HsRhCG* | [5] |  |
| p426-HsRhCG-GFP | p426MET25 *HsRhCG-(GA)_5_-GFP* | [6] |  |
| p426-HsRhCG^R202C^ | p426MET25 *HsRHCG^R202C^* | This study |  |
| p426-HsRhCG^R202C^-GFP | p426MET25 *HsRHCG^R202C^-(GA)_5_-GFP* | This study |  |
| p426-HsRhCG^A387T^ | p426MET25 *HsRHCG^A387T^* | This study |  |
| p426-HsRhCG^T45A^ | p426MET25 *HsRHCG^T45A^* | This study |  |
| p426-HsRhAG | p426MET25 *HsRhAG* | [5] |  |
| p426-HsRhAG-GFP | p426MET25 *HsRhAG-(GA)_5_-GFP* | [6] |  |
| p426-HsRhAG^I61R^ | p426MET25 *HsRHAG^I61R^* | This study |  |
| p426-HsRhAG^I61R^-GFP | p426MET25 *HsRHAG^I61R^-(GA)_5_-GFP* | This study |  |
| p426-HsRhAG^F65S^ | p426MET25 *HsRHAG^F65S^* | This study |  |
| p426-HsRhAG^F65S^-GFP | p426MET25 *HsRHAG^F65S^-(GA)_5_-GFP* | This study |  |
| p424 | pRS424MET25 *TRP1* | [4] |  |
| p424-HsRhCG | p424MET25 *HsRhCG* | This study |  |
| p424-HsRhCG^R202C^ | p424MET25 *HsRhCG^R202C^* | This study |  |
| p424-HsRhAG | p424MET25 *HsRhAG* | This study |  |
| YCpFL38 | CEN-ARS *URA3* | [7] |  |
| YCpMep2^N4Q^ | YCpFL38 *MEP2^N4Q^* | [8] |  |
| YCpMep2^N4Q,R211C^ | YCpFL38 *MEP2^N4Q,R211C^* | This study |  |

**References**

1. Marini AM, Soussi-Boudekou S, Vissers S, Andre B (1997) A family of ammonium transporters in Saccharomyces cerevisiae. Molecular and cellular biology 17: 4282–4293.

2. Mortimer RK, Johnston JR (1986) Genealogy of principal strains of the yeast genetic stock center. Genetics 113: 35–43.

3. Anderson JA, Huprikar SS, Kochian L V, Lucas WJ, Gaber RF (1992) Functional expression of a probable Arabidopsis thaliana potassium channel in Saccharomyces cerevisiae. Proceedings of the National Academy of Sciences of the United States of America 89: 3736–3740.

4. Mumberg D, Müller R, Funk M (1994) Regulatable promoters of Saccharomyces cerevisiae: comparison of transcriptional activity and their use for heterologous expression. Nucleic acids research 22: 5767–5768.

5. Marini AM, Matassi G, Raynal V, André B, Cartron JP, et al. (2000) The human Rhesus-associated RhAG protein and a kidney homologue promote ammonium transport in yeast. Nature genetics 26: 341–344.

6. Marini AM, Boeckstaens M, Benjelloun F, Chérif-Zahar B, André B (2006) Structural involvement in substrate recognition of an essential aspartate residue conserved in Mep/Amt and Rh-type ammonium transporters. Current genetics 49: 364–374.

7. Bonneaud N, Ozier-Kalogeropoulos O, Li GY, Labouesse M, Minvielle-Sebastia L, et al. (1991) A family of low and high copy replicative, integrative and single-stranded S. cerevisiae/E. coli shuttle vectors. Yeast 7: 609–615.

8. Marini AM, André B (2000) In vivo N-glycosylation of the mep2 high-affinity ammonium transporter of Saccharomyces cerevisiae reveals an extracytosolic N-terminus. Molecular microbiology 38: 552–564.
